# Preventing fraud in biomedical research

**DOI:** 10.3389/fcvm.2022.932138

**Published:** 2022-08-24

**Authors:** Elie Cogan

**Affiliations:** Department of Internal Medicine, CHIREC Hospitals and Université Libre de Bruxelles (ULB), Brussels, Belgium

**Keywords:** scientific misconduct, fraud, plagiarism, predatory journals, biomedical research

## Abstract

Scientific fraud represents, to varying degrees, an increasingly important part of medical literature and is estimated to make up nearly 20% of this literature. The increase in the number of articles accessible in preprint without peer review during the COVID-19 pandemic has led to an increase in the accessibility of fraudulent articles. In recent years, the viral increase in the number of predatory journals has contributed to polluting the scientific literature with articles whose content is unverifiable. Given the international nature of biomedical research, there is an urgent need to define unequivocally what is considered scientific fraud. In order to counter scientific misconduct, national and supranational procedures should be implemented to inform researchers at the beginning of their medical and biomedical training. Ethics commissions should implement local procedures for monitoring ongoing research. Finally, the fight against predatory journals requires information for researchers and the availability of tools to identify these journals.

## Introduction

### Definition and ethical considerations

Scientific fraud represents a particular form of embezzlement with multiple motivations—prestige, notoriety, vanity, secondary benefits sometimes financial gain. The psychological or even psychiatric component of the researchers involved cannot be ignored but has not been the subject of specific studies.

As Sox, editor of the Annals of Internal Medicine pointed out in 2006, “the scientific literature is a record of the search for truth. Publication of faked data diverts this search.” (1).

There is therefore an essential ethical responsibility for the entire scientific community not to pollute the medical literature with false data.

According to the Office of Research Integrity (ORI), research must be based on four main fundamentals: honesty, accuracy, efficiency, and objectivity (2).

Scientific research does not exclude error but requires a fully critical analysis of the data and results obtained. It is the fruit of collaboration and exchange of ideas and is nourished by the publication of results while requiring respect for the work of each individual and the fair attribution of merit in a highly competitive environment. It requires significant resources but cannot be distracted from perfect objectivity by the interests, even if implicit, of funders. Finally, it relies on peer review of work, a process where conflict of interest can be insidiously present.

### The quantitative dimension of fraud and retractions

Violations of scientific integrity are not so exceptional, as was already reported in a study published in Nature in 2005. A total of 2% of the authors admitted to having falsified results, and 30% admitted to possibly questionable scientific conduct (3).

In a survey carried out in five academic centers in Flanders in 2012, 15% of the 315 scientists interviewed admitted to having been directly involved in scientific misconduct in the last 3 years (4).

The problem is larger than one might imagine and could be as high as 20% of publications (5).

Chambers et al. examined retracted articles among articles published between 1985 and 2018 in the field of gynecology and obstetrics. A total of 176 articles were retracted. The most frequent cause was plagiarism. The retraction rate increased over time and journals with high impact factors were not spared (6).

For publications in the cardiovascular field, 459 retractions could be documented for articles published between 1975 and 2020. The percentage of retractions increased steadily until 2015 and then decreased. The average time between publication and retraction was 1.4 years but tended to decrease over time (7).

The main reason for the retraction of publications in the biomedical field is scientific misconduct, which represents more than 67% of the causes of retractions, including fraud or suspected fraud (i.e., data fabrication and/or falsification of the data), duplicate publication, and plagiarism (8).

The recent COVID-19 pandemic has contributed to an explosion in the dissemination of non-peer-reviewed research results, thus increasing the risk of dissemination of irrelevant scientific information, sometimes resulting from fraudulent practices (9). It should be recognized, however, that these preprint servers (e.g., bioRxiv, medRixiv) clearly warn the reader that preprints are preliminary reports not yet certified by peer review and that this information should not be disseminated in the media as established scientific information. It is disappointing that the media do not respect this kind of warning.

This clearly poses the problem of controlling the dissemination of scientific information not validated by a peer review process, which is sometimes picked up by the media and disseminated to the public, irrespective of the warnings clearly expressed on the homepage of the preprint servers.

### Research misconduct classification

Misconduct of research integrity can be grouped into four broad categories: breaches in obtaining scientific knowledge, in collaboration and publication, in obtaining research funding, or in providing scientific expertise to others.

The seriousness of scientific misconduct is directly related to the harm done to society, to science, to the institution to which the breaching party belongs, and also to other scientists who might be the victims.

A high-level British Medical Journal and the Committee on Publication Ethics (BMJ/COPE) meeting was organized in London 10 years ago in order to establish a consensus statement on research misconduct (10).

It is important to emphasize that the behavior of a researcher who deviates from ethical and scientific standards should be considered scientific misconduct, whether or not the deviation is intentional. It was stated that “research misconduct includes fabrication, falsification, suppression, or inappropriate manipulation of data; inappropriate image manipulation; plagiarism; misleading reporting; redundant publication; authorship malpractice such as guest or ghost authorship; failure to disclose funding sources or competing interests; misreporting of funder involvement; and unethical research (for example, failure to obtain adequate patient consent)” (10).

However, the definitions of research misconduct are not uniform in national policies as reported in a large and systematic survey performed in 2014 on misconduct policies in the 40 top countries in research and development (11). Fabrication, falsification, and plagiarism were included in the definition of scientific misconduct in 100% of the 22 countries (55%) that had a national misconduct policy. However unethical authorship was just mentioned in only 54.6% of the misconduct definitions and unethical publication practices or unethical peer review practices in about one-third of the countries (11).

## An analysis of the causes of scientific misconduct

A thorough analysis of the causes of violations of scientific integrity is needed in order to develop preventive procedures.

### Pressure to publish

Pressure to publish is a major cause of scientific misconduct.

Money is one of the most important factors behind scientific fraud. Obtaining the funds needed to carry out research projects is directly linked to one's own scientific output. A large number of researchers find themselves under pressure to publish their results as quickly as possible or risk not having their contracts renewed. The expression “publish or perish” describes this race to publish with the real risk of encouraging fraud either by manipulating data or by purely fabricating the data needed for publication.

But the pressure to publish can also come from ethically reprehensible heads of laboratories or departments. For example, one study showed that one-fifth of young people who do research are pressured by their supervisors to publish non-reproducible results, and one-third say they have had to steer their research toward results desired by their supervisor (12).

### Psychological issues

Pressure to publish is associated with psychological stress and appears to be a key factor associated with scientific misconduct (4).

One cannot ignore either the psychiatric dimension present in a certain number of fraudsters in the biomedical field. Their psychological profile is dominated by an inflation of the ego with a search for omnipotence. The narcissistic dimension is in the foreground with a constant search for recognition. This includes scientific recognition but also social recognition.

The psychological or even psychiatric aspects in the biomedical field are shared with what is found in the industrial or financial field. In fact, fraud in the scientific field has points in common with any form of swindling with three conditions regularly found which constitute the triangle of fraud: motivation (financial pressure, search for grants); rationalization (justification of the fraud), and opportunity, i.e., situations allowing the realization of the fraud (13).

### Predatory journals

Publications in so-called predatory journals have increased dramatically in recent years, contributing to the dissemination of false sciences.

Incentives to publish in predatory journals that offer the assurance of publication in a moderately paid open access journal are a new type of temptation to publish the results of dubious research without the risk of a serious peer review (14).

Predatory journals contribute to the flooding of databases with fraudulent publications when articles are not properly peer-reviewed and the validity of the data is not checked.

The main characteristic of this type of publication is the publication of scientific content, without a peer review process, in a relatively short time from submission to publication, for a fairly large sum of money.

Predatory journals employ many seemingly respectable strategies and techniques to attract the interest of naïve young researchers who would be eager to publish quickly, in order to get promoted.

Beall is considered an expert in the hunt for predatory journals. Thus, he aptly points out that predatory publishers are corrupting open access journals that exploit the author-pays model undermining scholarly publishing and encouraging unethical behavior by scientists (15).

Most young and junior researchers, but also respectable researchers, actively publish in fraudulent or fictitious journals in order to enhance their curriculum vitae and increase their publication output to obtain jobs, finance further studies, and qualify for grants and promotions in academic careers.

As a result, several predatory journals have begun to enter credible databases, such as, for example, PubMed, SCOPUS, and Web of Science.

While fabricating or falsifying data is clearly the responsibility of researchers and/or their chiefs, the dissemination of false science in predatory journals is primarily the responsibility of the editors of these journals and even the organizations on which they depend.

This distinction is important, particularly when it comes to considering ways of combating these abuses.

## Prevention of scientific fraud

### General considerations

Given the international nature of biomedical research with co-authors affiliated with institutions in different countries, there is an urgent need to define common standards for scientific fraud.

Apart from the primary responsibility of the researchers, the responsibility of the institutions employing the researchers must also be stressed. This obviously concerns university institutions and research laboratories but also, in particular with regard to clinical studies and hospitals, whether or not they are linked to a university.

The responsibility of journal editors cannot be evaded. The importance of the choice of reviewers and careful and scrupulous control of the articles submitted is fundamental. The problem posed by predatory journals is increasingly important since quality control of scientific production published in these journals is far from guaranteed.

Finally, bibliographic databases should be consulted with confidence. However, it appears that an increasing number of articles included in widely consulted databases such as PubMed are found to be fraudulent.

### Education and information: Initial steps in prevention

The first step in fraud prevention is to implement an information program on good clinical practice and research quality criteria. This education should start in the early years of the medical or biomedical sciences curriculum.

A significant number of mistakes can be considered unintentional. These are errors that do not necessarily fall under the label of gross fraud. These “honest” errors are due to methodological misconceptions (lack of documentation research, inappropriate methods, statistical errors, inappropriate samples). Targeted teaching is needed to avoid these types of errors, which are unfortunately not always identified by reviewers. The information should include recommendations on publication rules, in particular, authorship or co-authorship of an article.

The role of supervisors, laboratory directors, or heads of departments in clinical research should not be underestimated.

It is also a matter of informing not only young researchers but also more experienced ones that fabrication of data, falsification of data, and plagiarism are all considered to be intentional fraud and are punishable with serious sanctions up to dismissal from the institution.

Finally, warning about predatory journals is an important element in the education of young researchers (see below).

### Protocol control committees

The approval of research projects, in particular clinical research, depends on an in-depth analysis of the protocol by an ethics committee, either academic (under a medical faculty) or linked to the hospital, most often an academic hospital associated with a medical faculty.

Once the research project is accepted, most institutions lack control procedures to monitor the application of the protocol as validated by the ethics commission. It would therefore be necessary to impose within these structures the creation of a supervisory commission attached to the ethics commission, whose aim would be to validate the raw data generated throughout the experimentation. In the case of clinical studies, it would also be appropriate to ensure that patients have really met the conditions for selection in the study. The composition of this monitoring committee should include research experts from the university but also independent persons from outside the institution or university.

### Controlling predatory journals

As mentioned above, predatory journals pose a growing risk of polluting the scientific literature.

The first step is to inform researchers of the definition of a predatory journal. One of the first elements that should alert researchers is the solicitation to send an article to an open access journal that asks for significant publication rights with the guarantee of publication.

These journals do not bother to look for plagiarism and generally, there is no real peer review.

It is therefore a question of making tools available to identify the predatory nature of a scientific journal (16).

A list of alleged journals and publishers associated with predatory journals is available online as Beall's list (available at https://beallslist.net). This website is a copy of Beall's list of predatory publishers and journals. It was retrieved from the cached copy on 15 January 2017.

The initial list includes over 1,000 journals and publishers (!) covering all disciplines not limited to the biomedical sciences. These are journals that are suspected of being predatory, although there is no way to be certain. The aim would be to draw a researcher's attention to the possible predatory nature of the journal. This list is nonetheless open to criticism as it is based essentially on Jeffrey Beal's intuition and has been the subject of serious criticism (17).

Cabells' Predatory Reports is a paid subscription service comprising a database of misleading and predatory journals and a database of “verified and reputable journals,” with details of their acceptance rates and percentages of invited articles (18).

Compass to publish, currently in beta version, is a tool being developed by the University of Liege in Belgium to warn about the authenticity of a journal or the predatory nature of a journal or publisher. It is an online questionnaire that ultimately indicates the probability that the journal in question should be considered suspicious. This tool, currently in French, is available at https://app.lib.uliege.be/compass-to-publish/.

It is therefore essential that a recognized international body establishes a list of predatory journals and publishers and that these journals are automatically excluded from scientific databases such as PubMed, Scopus, Web of Science, etc.

This official list should be known to researchers, publishers, and of course reviewers. *Ad hoc* software should be developed for researchers, reviewers, and publishers to exclude from the list of references of submitted manuscripts those that correspond to well-defined predatory journals (19).

This official list should allow all databases collecting biomedical literature to be purged of predatory journals (19).

At the academic level, articles published in these journals should no longer be taken into account, but researchers who have published in those journals clearly identified as predatory should be admonished and even sanctioned.

### Promoting the role of the reviewer

As mentioned above, the role of reviewers is decisive not only in rejecting articles that do not reach a sufficient scientific level but also in detecting fraud.

There is currently no reviewer status. They are volunteers and are selected by the publishers on the basis of their reputation and expertise in a given field based on their publications. We recommend that specific training be organized for reviewers and that they be paid for their work. It would also be important to establish evaluation processes for reviewers under the responsibility not only of publishers but of independent bodies and even universities.

In addition, reviewing for a journal should be valued in the curriculum vitae in the same way as being a thesis promoter, for example.

### Developing investigative structures

The code of conduct should mandate among other things the following: the setting up of effective systems to prevent and detect misconduct, including the protection of whistleblowers and the duty of researchers to report misconduct.

Proper investigation of allegations of research misconduct, including as a minimum the reporting of results of investigations to a national advisory and oversight body such as the UK Research Integrity Office (UKRIO) or the ORI, which depends on the U.S. Department of Health and Human Services.

University institutions but also hospitals involved in clinical research must register and subscribe to such a body. The advisory, support, and oversight roles of UKRIO should be enhanced, and it should be properly and securely financed (10).

As the dissemination of scientific discoveries no longer knows borders, scientific fraud is more than ever an international concern. National and international fraud prevention policies are essential to monitoring research integrity, although many institutions have developed their own policies. Indeed, while institutional procedures play an essential role in the prevention and suppression of fraud, national policies are also important to ensure the consistent promulgation and enforcement of ethical standards (11).

Misconduct policies can play a crucial role in preventing and policing research misconduct. Policies typically include a definition of misconduct as well as procedures for investigating and adjudicating misconduct (11).

A national misconduct policy could be defined as a law, regulation, or government funding agency policy operating at the national level that addresses research misconduct.

In France, on the initiative of the Ministry of Higher Education, Research, and Innovation, a decree on compliance with the requirements of scientific integrity by public establishments contributing to the public research service and foundations recognized as being of public utility whose main activity is public research was published in December 2021.

## Conclusion

The fight against scientific fraud involves all actors involved in biomedical research.

Since fraud concerns not only researchers but also research structures, reviewers, and press groups involved in the dissemination of research, the solution can only be systemic. The problem of scientific fraud goes beyond the pollution of scientific truth but can constitute a real danger in terms of public health. A regulation of scientific integrity must be addressed by national governments in charge of higher education and public health in the framework of a supranational regulation.

## Author contributions

The author confirms being the sole contributor of this work and has approved it for publication.

## Conflict of interest

The author declares that the research was conducted in the absence of any commercial or financial relationships that could be construed as a potential conflict of interest.

## Publisher's note

All claims expressed in this article are solely those of the authors and do not necessarily represent those of their affiliated organizations, or those of the publisher, the editors and the reviewers. Any product that may be evaluated in this article, or claim that may be made by its manufacturer, is not guaranteed or endorsed by the publisher.
